# Defining the Predicted Protein Secretome of the Fungal Wheat Leaf Pathogen *Mycosphaerella graminicola*

**DOI:** 10.1371/journal.pone.0049904

**Published:** 2012-12-07

**Authors:** Alexandre Morais do Amaral, John Antoniw, Jason J. Rudd, Kim E. Hammond-Kosack

**Affiliations:** 1 Embrapa LabEx Programme, Rothamsted Research, Harpenden, Herts, United Kingdom; 2 Department of Plant Biology and Crop Science, Rothamsted Research, Harpenden, Herts, United Kingdom; Universidade de Sao Paulo, Brazil

## Abstract

The Dothideomycete fungus *Mycosphaerella graminicola* is the causal agent of Septoria tritici blotch, a devastating disease of wheat leaves that causes dramatic decreases in yield. Infection involves an initial extended period of symptomless intercellular colonisation prior to the development of visible necrotic disease lesions. Previous functional genomics and gene expression profiling studies have implicated the production of secreted virulence effector proteins as key facilitators of the initial symptomless growth phase. In order to identify additional candidate virulence effectors, we re-analysed and catalogued the predicted protein secretome of *M. graminicola* isolate IPO323, which is currently regarded as the reference strain for this species. We combined several bioinformatic approaches in order to increase the probability of identifying truly secreted proteins with either a predicted enzymatic function or an as yet unknown function. An initial secretome of 970 proteins was predicted, whilst further stringent selection criteria predicted 492 proteins. Of these, 321 possess some functional annotation, the composition of which may reflect the strictly intercellular growth habit of this pathogen, leaving 171 with no functional annotation. This analysis identified a protein family encoding secreted peroxidases/chloroperoxidases (PF01328) which is expanded within all members of the family *Mycosphaerellaceae*. Further analyses were done on the non-annotated proteins for size and cysteine content (effector protein hallmarks), and then by studying the distribution of homologues in 17 other sequenced Dothideomycete fungi within an overall total of 91 predicted proteomes from fungal, oomycete and nematode species. This detailed *M. graminicola* secretome analysis provides the basis for further functional and comparative genomics studies.

## Introduction

Plant pathogenic fungi and oomycetes secrete an arsenal of proteins and metabolites during infection of their hosts. Protein secretion in eukaryotes can occur via a classical or non-classical route [1].Whilst it is apparent that certain fungi secrete proteins via a non-classical route [2], the mechanism is to date unknown and no clear molecular signatures for this exist. Classical secretion on the other hand is mediated through the presence of an N-terminal signal peptide which establishes the basis of transit though the endoplasmic reticulum and golgi systems on route to secretion from the cell. Some of the proteins secreted by plant pathogenic fungi have assignable functions and may include a diversity of hydrolytic enzymes which attack the plant cell wall or degrade other complex carbon or nitrogen sources, for example secreted proteases and glucanases. However many secreted proteins lack any obvious functional motifs and are frequently too small to encode catalytic activities. These small secreted proteins are often referred to as effectors (or candidate effectors) and are believed to function to counteract or suppress host defences and/or mask detection by the plant immune systems. In many cases plants have evolved the capability to recognise either directly or indirectly these effectors through disease resistance (R) proteins and/or guardee proteins giving rise to the widely accepted “gene-for-gene” model of effector triggered immunity [3]. This frequently activates a particular defence response involving highly localised cell death, termed hypersensitive cell death, which is particularly effective against biotrophic pathogens. For fungi which ultimately have a necrotrophic lifestyle, some of the small secreted effector proteins have been shown to target plant susceptibility (S) proteins encoded by homologues of resistance genes. In these cases, the resulting S protein – effector interaction triggers widespread HR for the benefit of the pathogen. This remarkable “hijack” of plant disease resistance mechanism has been termed an “inverse gene-for-gene” interaction [4], [5]. Both examples highlight that pathogens require a very specific and selective effector repertoire which enable them to infect their often restricted range of host plants and/or particular plant tissues, to then cause disease and finally complete their lifecycle through asexual/sexual sporulation.

*Mycosphaerella graminicola* (anamorph *Septoria tritici*, recently renamed *Zymoseptoria tritici* (Desm.) Quaedvlieg & Crous, *comb. nov.* [6]), is the causal agent of Septoria tritici blotch (STB) disease [7]. STB currently ranks as one of the most economically important diseases of wheat in the UK and Western Europe, and a threat to yields worldwide [8]. *M. graminicola* exhibits both host and tissue specificity, infecting only the leaves of wheat (*Triticum* spp) plants. Following leaf penetration through stomata, the hyphae then grow intercellularly throughout the leaf mesophyll cell layer for at least seven days post inoculation in the absence of any visible symptoms of disease [9]. Typically 8–10 days after inoculation disease lesions begin to form on susceptible plants [9]. This transition is associated with induction of host defence responses sharing characteristics with a hypersensitive response and involving differential regulation of plant defence signalling pathways [10], [11]. These events culminate in loss of control of cell permeability resulting in leakage of nutrients from dying plant cells into the intercellular (apoplastic) spaces. This coincides with an exponential increase in fungal growth rate. Asexual sporulation structures (pycnidia) then form in the sub-stomatal cavities of necrotic leaf tissues. The asexual pycnidiospores extrude through stomatal openings and are dispersed via rain splash throughout the crop canopy, giving rise to polycyclic infections [9]. This strictly intercellular (or apoplastic) growth lifestyle of *M. graminicola* is a characteristic shared with other *Mycosphaerellaceae* plant pathogens, which distinguishes them from various other globally important fungal plant pathogens including *Fusarium* spp and *Magnaporthe* spp which penetrate host cells during at least one stage of their infection cycle [8].

The sequenced genome of *M. graminicola* isolate IPO323 was recently published [12]. The current gene model prediction (11,035) was greatly aided by the production of over 50,000 expressed sequence tags deriving from various libraries. The genome spans 21 chromosomes, although it has been established that the eight smallest of these can be lost without affecting the ability of the fungus to cause disease [12], [13], and these are now referred to as dispensable chromosomes (the dispensome). First analysis of the constituents of this genome indicated that *M. graminicola* may have less potential for degrading plant cell walls due to possessing a limited number of genes encoding appropriate hydrolytic enzymes. Conversely several protease encoding gene families were expanded suggesting that host cell protein degradation might be an important source of fungal nutrition during infection [12]. However it was unclear how many of these putative proteins were likely to reside in the overall protein secretome, as to date no proteome based analyses have been published for this organism.

It is assumed that many fungal effectors are most likely to be soluble, extracellular secreted proteins that do not become cross-linked into the fungal cell wall [14]. It was also unclear, until recently, to what extent *M. graminicola* might rely on the deployment of effectors to either facilitate the initial symptomless growth phase (evasion or suppression of plant defence), and/or to trigger host cell death underlying the appearance of disease lesions [15]. However we recently identified a key role for at least one fungal predicted secreted protein effector in facilitating the symptomless phase of leaf infection through its activity in suppressing chitin-mediated plant defences. This effector, referred to as Mg3LysM, contains three predicted LysM domains which function to bind chitin fragments and prevent the elicitation of plant defences [16]. Mg3LysM and other MgLysM effectors were identified on the basis of their homology to CfECP6, the first LysM effector identified in plant pathogenic fungi, via a purely biochemical route, from the tomato leaf mold fungus *Cladosporium fulvum* [17], [18]. The functional conservation of the unique LysM effector activities in several plant pathogenic fungi irrespective of host range highlights the power of comparative genomics for putative fungal effector discovery. Despite the first evidence for secreted effector protein function during symptomless colonisation, it remains wholly unclear how many putative secreted protein effectors *M.graminicola* possesses, and how many might function in suppressing early defences or in subsequently triggering defences to support the activation of host cell death signalling and to facilitate the necrotrophic growth phase and asexual sporulation.

The computationally predicted classical secretomes of phytopathogenic fungi and oomycetes present powerful tools to compare and contrast between species with different host and tissue specificities as well as nutritional preferences. For filamentous fungi, predicted and well annotated classical secretomes have been deciphered by purely bioinformatics approaches for the basidiomycetes *Ustilago maydis* [19] and *Puccinia graminis* [20] as well as the ascomycete *Fusarium graminearum* [21]. The three species, like *M. graminicola* infect one or more cereal host plant species but have differing tissues specificities and nutritional lifestyles. To further analyse the predicted classical secretome of *M. graminicola* we have performed a rigorous bioinformatics analysis. The results of these analyses are reported here, together with a 91 member interspecies comparison, involving 17 additional Dothideomycete fungi, most of which are plant pathogens including 5 other *Mycosphaerellaceae* species, in addition to other fungi with contrasting lifestyles, oomycete species and plant pathogenic nematodes. This comprehensive analysis provides a basis for further candidate effector protein discovery via follow-up genomics based approaches.

## Results

### The total predicted and refined secretome of *M. graminicola* isolate IPO323

**Figure 1:**
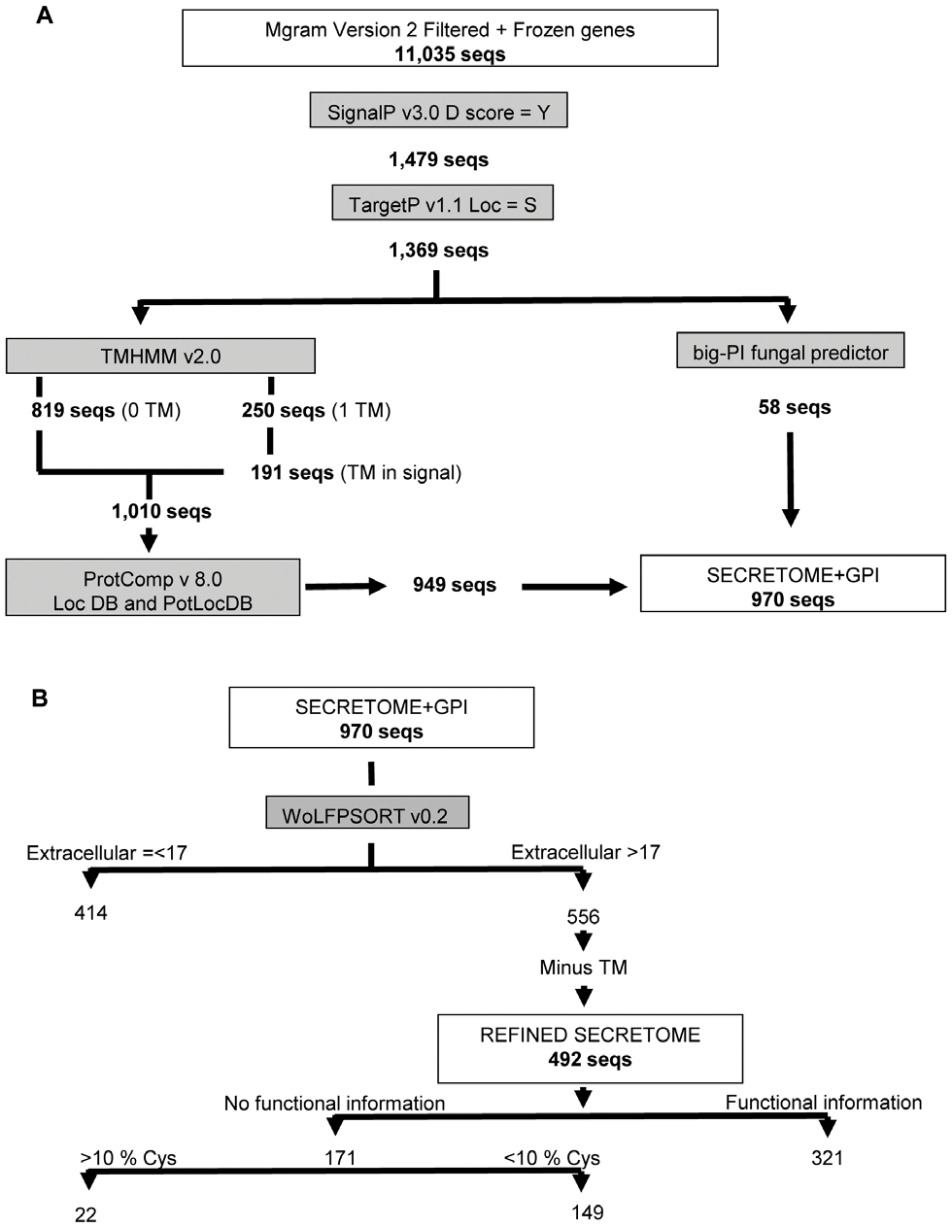
The two stage automated secretome prediction pipeline of *M. graminicola*. (**A**) Stage 1 used to predict the total secretome and (**B**) stage 2 used to predict the refined secretome. In stage 1, 37 of the 949 sequences are also predicted to be GPI-anchor proteins.

In the current study we analysed the combined filtered and frozen gene call (11,035 unique proteins) of version 2 the *M. graminicola* genome in two phases. In the first stage (Figure 1A), designed to predict all possible secreted proteins (the “total” secretome), SignalP and TargetP were used to identify secreted proteins with signal peptides (1,369 proteins). Fifty-eight of these proteins were subsequently predicted to contain GPI anchors. After removal of the signal peptide sequence from each sequence, any mature proteins that contained a transmembrane domain (TM) were excluded. We then used the ProtComp software to exclude proteins that were probably not located in the extracellular space. This predicted the total secretome for *M. graminicola* and contained 970 proteins (including those with GPI anchors). This represents 8.8% of the total current predicted *M. graminicola* protein models. For completeness, and to assist with follow up comparative analyses, the results for the predicted total secretome with the larger size of 970 genes arising from stage 1 of the analysis are presented in Tab 1 in File S1.

Stage 2 (Figure 1B), designed to identify a subset of proteins with an even higher probability of being secreted (the “refined” secretome), applied more stringent conditions to further analyse this set of proteins. A second software package (WoLF PSORT) that also predicts the eventual location of proteins was used to define proteins that have a high probability of being secreted into the extracellular spaces (extracellular score >17). This resulted in a reduced set of 556 secreted proteins (Tab 2 in File S1). After this stage, the proteins that lacked a methionine in the first position (9 proteins) were removed (Tab 3 in File S1) and the 55 proteins with a TM domain predicted within the signal peptide sequence were also removed. This resulted in the final prediction for the refined secretome for *M. graminicola* (Tab 4 in File S1) and contained 492 proteins (i.e. 4.5% of the total current predicted *M. graminicola* protein models). Overall, the predicted mature protein length for the refined secretome ranged in size from 33 to 1,369 amino acids. We next downloaded from the JGI genome portal the annotation and functional classification (where available) for the 492 secreted proteins present in the MG 2 gene call (http://genome.jgi-psf.org/Mycgr3/Mycgr3.home.html). This information is also presented in Tab 4 in File S1. Out of these 492 proteins, 321 (65%) possessed information on protein function (Tab 5 in File S1, columns PFAM, KOG, CDD, jgi-domains, jgi-go_info, jgi-kog_info and definition) whilst 171 (35%) (Figure 1B, Table 1) were described as hypothetical or conserved hypothetical (Tab 6 in File S1).

**Table 1:** Distribution of secreted proteins throughout the 21 chromosomes of *Mycosphaerella graminicola.*

Chr	Size (nt)^1^	Proteins		Secreted proteins per Mb	Annotation^2^		Unique^2^
		Total	Secreted		Yes	No	
1	6,088,797	1,998	81	13.3	57 (70)	24 (30)	12 (15)
2	3,860,111	1,149	51	13.2	27 (53)	24 (47)	9 (18)
3	3,505,381	1,078	55	15.7	37 (67)	18 (33)	6 (11)
4	2,880,011	830	35	12.2	29 (83)	6 (17)	7 (20)
5	2,861,803	786	35	12.2	25 (71)	10 (29)	4 (11)
6	2,674,951	695	32	12.0	18 (56)	14 (44)	6 (19)
7	2,665,280	770	40	15.0	26 (65)	14 (35)	6 (15)
8	2,443,572	702	30	12.3	23 (77)	7 (23)	8 (27)
9	2,142,475	609	30	14.0	18 (60)	12 (40)	3 (10)
10	1,682,575	521	26	15.5	18 (69)	8 (31)	5 (19)
11	1,624,292	490	24	14.8	15 (62)	9 (38)	5 (21)
12	1,462,624	415	30	20.6	15 (50)	15 (50)	7 (23)
13	1,185,774	338	23	19.5	13 (56)	10 (44)	7 (30)
14	773,098	114	0	-	-	-	-
15	639,501	86	0	-	-	-	-
16	607,044	88	0	-	-	-	-
17	584,099	78	0	-	-	-	-
18	573,698	64	0	-	-	-	-
19	549,847	87	0	-	-	-	-
20	472,105	79	0	-	-	-	-
21	409,213	58	0	-	-	-	-
Total	39,686,251	11,035	492		321	171	85

1 – ref [12];

2 - In parentheses, %.

### EST expression support for the predicted refined secretome

Prior to sequencing the genome of the reference isolate IPO323, 27,000 ESTs were produced from 10 libraries using this isolate [22]. In addition, approximately 4,000 ESTs were produced from a UK field isolate [23]. All these ESTs are displayed and retrievable from the JGI genome website (http://genome.jgi-psf.org/Mycgr3/Mycgr3.download.ftp.html). We checked each of the 492 genes for any level of EST support displayed on the JGI genome browser. This led to a final total of 262 which have some level of EST support (Tab 4 in File S1). The remainder currently have none. Therefore to date approximately 53% of the refined secretome has EST support. However, this value is likely to be an underestimate of the proportion that encodes transcribed genes. For example, there are currently no ESTs aligned to the *M. graminicola* gene *MgNLP*, shown by qPCR to vary in expression during fungal growth in liquid culture and across phases of plant infection [24]. In summary therefore, this indicates that at least a minimum of 53% of the predicted refined secretome are actively transcribed genes in at least one biological situation. More specifically we identified 75 predicted proteins which had EST support from both *in vitro* culture and plant infection based libraries (see later). Whereas 127 had EST support solely from *in vitro* libraries and 60 had support only from plant infection libraries.

### The distribution of the refined secretome throughout the genome sequence

**Figure 2:**
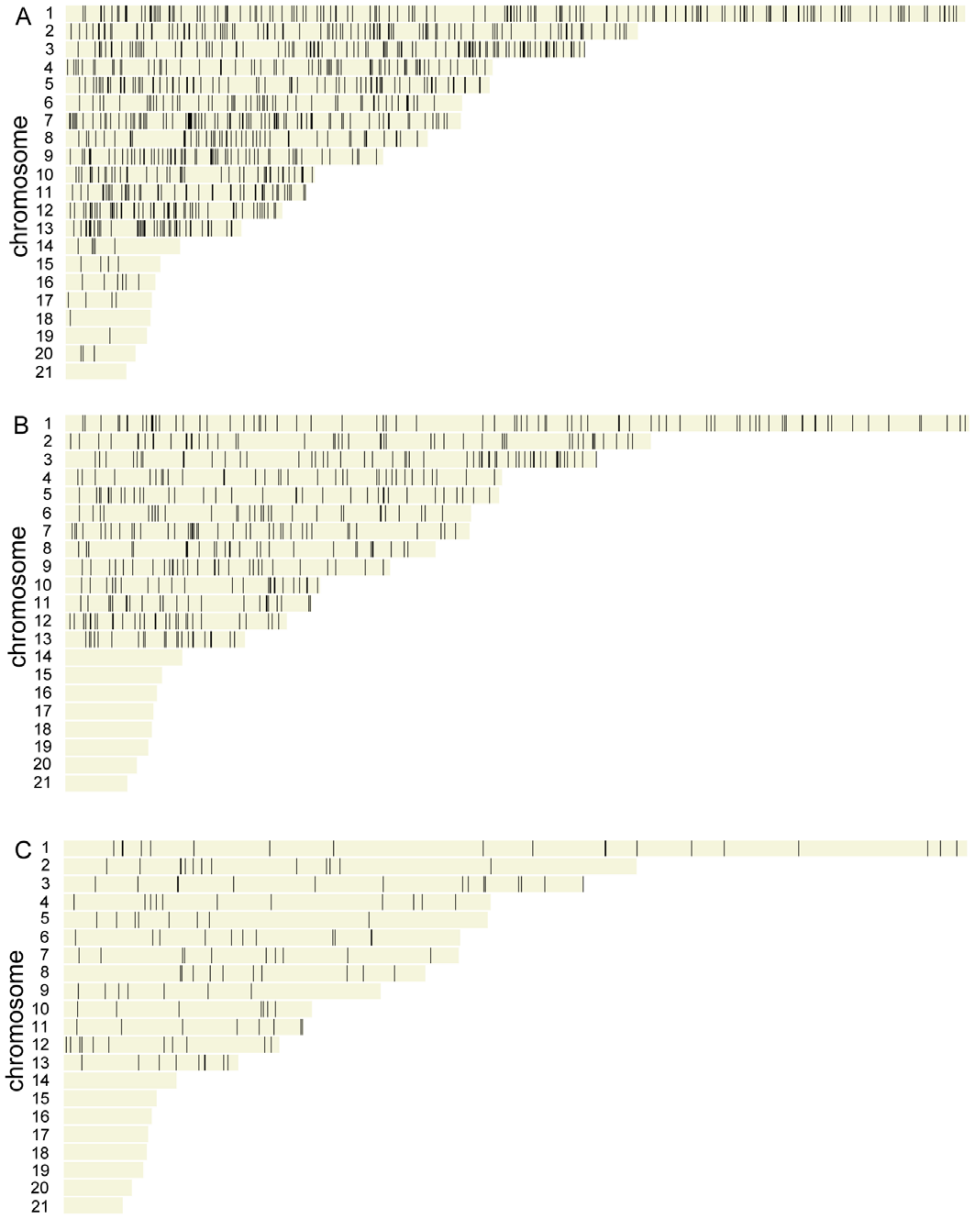
The genes predicted to code for the secretome of *M. graminicola* displayed over the 21 chromosomes. (**A**) The total 970 genes arising from stage 1 of the analysis. (**B**) The refined 492 genes arising from stage 2 of the analysis. (**C**) The 85 genes found so far to be *M. graminicola* specific.

To reiterate, the sequenced reference isolate of *M. graminicola* (IPO323) possesses 21 chromosomes with the smallest eight chromosomes being dispensable for plant infection [13]. Whilst 24 of the total set of 970 genes predicted to code for secreted proteins (Fig. 2A) reside on these dispensable chromosomes, none of the refined 492 predicted proteins reside on them.

The location of the genes coding for the refined secretome proteins was explored over the 13 core chromosomes (Fig. 2B). The predicted secreted protein coding gene density (*ie*, number of secreted proteins per Mb) was comparable for chromosomes 1 through 11, with an average of 13.7 genes per Mb (Table 1). By contrast, on the two shortest core chromosomes, 12 and 13, the density was slightly higher at 20.6 and 19.5 genes per Mb, respectively (Table 1).

### Identification of predicted secreted proteins with enzymatic functions

**Figure 3:**
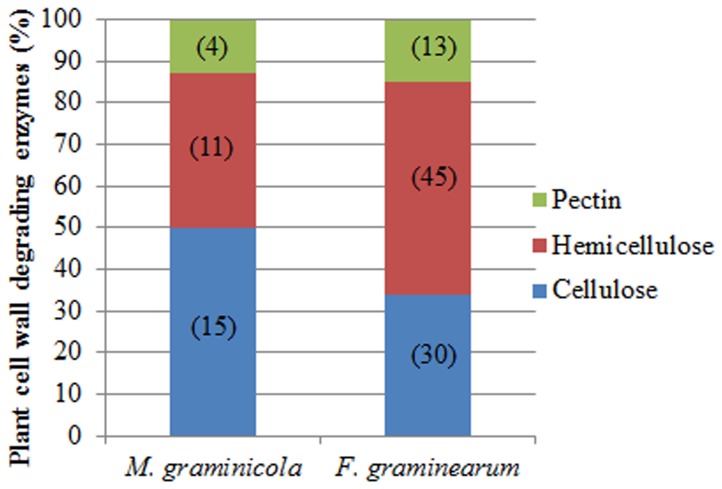
Comparison of the distribution of functional categories (main PCWDEs) identified in the predicted secretomes of *M. graminicola* and *F. graminearum*. The various enzyme classes are given along with the actual number of enzymes in each class (in brackets).

Among all 321 predicted secreted proteins with known or presumed functions, at least 64 are suggested to be involved in the degradation of polysaccharides (Table 2 and Tab 7 in File S1). Thirty-nine proteins have functions related to protein degradation and 29 are implicated in the modification of lipids (Tables 3 and 4). The genome sequencing of isolate IPO323 identified 184 glycoside hydrolases in total. Of the 184 predicted glycoside hydrolases, 54 are present in the refined secretome (Table 2) with 22 of these proteins predicted to be directly involved in modifying the plant cell wall (cellulose, hemicellulose and pectin). These secreted plant cell wall degrading enzymes (PCWDEs) comprise 28 glycoside hydrolases, 6 esterases and 1 pectate lyase (Table 5 and Tab 8 in File S1). A direct comparison with the predicted refined secretome of the wheat ear attacking fungus *Fusarium graminearum* [21] highlights a drastically reduced overall complement of secreted PCWDE's in *M. graminicola* (Figure 3) as initially alluded to by previous analysis of the total genome content [12]. All five predicted cellulases identified in the genome of *M. graminicola* [12] were found to be present in the refined secretome. In addition, the refined secretome possessed only two members of the Glycoside hydrolase family 61 (GH61), with protein Ids 33254 and 103512. Although still poorly studied, GH61 functions have been associated with improving cellulose breakdown when acting alongside cellulases [25]–[27], and members of this protein family were also found in reduced numbers in the genome of *M. graminicola* as compared to other plant pathogens [12]. For comparison, in *F. graminearum* at least 11 members of GH61were found in the predicted refined secretome [21]. *M. graminicola* is also predicted to secrete at least four cutinases (protein Ids 43394, 68483, 77282 and 99331) (Table 4). Finally, we identified 28 predicted secreted proteins with functions relating to the modification of the fungal cell wall (Tab 9 in File S1), including 2 members of the glycoside hydrolase family 18 and one member of glycoside hydrolase family 75 where the substrate is predicted to be chitin.

**Table 2:** The sub-set of *M. graminicola* genes that code for secreted proteins involved in the degradation of polysaccharides.

Annotation	#id-JGI (a.a. size)	Functional annotation	PFAM
Tannase	46238 (521), 49510 (552), 51439 (516)	Hydrolysis of carbohydrate esters	PF07519
Esterase PHB depolymerase	95636 (315), 106075 (283)	Hydrolysis of carbohydrate esters	PF10503
Pectinesterase	66866 (314)	Hydrolysis of carbohydrate esters	PF01095
α-L-arabinofuranosidase B, catalytic/GH54	70396 (309)	Hydrolysis of α -1,2-, α -1,3- and α-1,5-L-arabinofuranosidic linkages	PF09206
α-L-arabinofuranosidase C-terminus//GH51	71466 (605), 111130 (670)	Hydrolysis of nonreducing terminal α-L-arabinofuranosidic linkages	PF06964
Glycoside hydrolase family 1	49899 (603)	Hydrolysis of glycosidic bonds	PF00232
Glycoside hydrolase family 2	87705 (606)	Hydrolysis of glycosidic bonds	PF02836
Glycoside hydrolase family 3	42323 (846), 42620 (896), 44498 (802), 64142 (848), 71284 (767), 85505 (751), 99970 (861)	Hydrolysis of glycosidic bonds	PF00933, PF01915
Glycoside hydrolase family 5	88889 (400), 106779 (397)	Hydrolysis of glycosidic bonds	PF00150
Glycoside hydrolase family 7	100252 (426)	Hydrolysis of glycosidic bonds	PF00840
Glycoside hydrolase family 10	61141 (328)	Hydrolysis of glycosidic bonds	PF00331
Glycoside hydrolase family 11	60105 (207)	Hydrolysis of glycosidic bonds	PF00457
Glycoside hydrolase family 12	105871 (223)	Hydrolysis of glycosidic bonds	PF01670
Glycoside hydrolase family 13/Alpha-amylase	65440 (498), 86748 (477)	Hydrolysis of glycosidic bonds	PF09260, PF00128
Glycoside hydrolase family 15/Carbohydrate-binding module	42503 (593)	Hydrolysis of glycosidic bonds	PF00723, PF00686
Glycoside hydrolase family 16	37166 (264), 74453 (280), 83802 (351), 102047 (421)	Hydrolysis of glycosidic bonds	PF00722
Glycoside hydrolase family 17	107711 (291)	Hydrolysis of glycosidic bonds	PF00332
Glycoside hydrolase family 18	86391 (381), 99379 (356)	Hydrolysis of glycosidic bonds	PF00704
Glycoside hydrolase family 20	100496 (557)	Hydrolysis of glycosidic bonds	PF00728, PF02838
Glycoside hydrolase family 28	77196 (470)	Hydrolysis of glycosidic bonds	PF00295
Glycoside hydrolase family 30	92113 (460)	Hydrolysis of glycosidic bonds	PF02055
Glycoside hydrolase family 31	31465 (742)	Hydrolysis of glycosidic bonds	PF01055
Glycoside hydrolase family 31/Carbohydrate-binding module	111695 (971)	Hydrolysis of glycosidic bonds	PF01055, PF00686
Glycoside hydrolase family 32	74191 (545)	Hydrolysis of glycosidic bonds	PF00251
Glycoside hydrolase family 36	110289 (790)	Hydrolysis of glycosidic bonds	PF02065
Glycoside hydrolase family 37	48841 (621)	Hydrolysis of glycosidic bonds	PF01204
Glycoside hydrolase family 43	75584 (652), 96505 (365), 105323 (300)	Hydrolysis of glycosidic bonds	PF04616
Glycoside hydrolase family 45	76589 (221)	Hydrolysis of glycosidic bonds	PF02015
Glycoside hydrolase family 47	47168 (538), 54752 (490)	Hydrolysis of glycosidic bonds	PF01532
Glycoside hydrolase family 53	51381 (327)	Hydrolysis of glycosidic bonds	PF07745
Glycoside hydrolase family 61	33254 (299), 103512 (369)	Hydrolysis of glycosidic bonds	PF03443
Glycoside hydrolase family 62	68922 (302)	Hydrolysis of glycosidic bonds	PF03664
Glycoside hydrolase family 65	69330 (992)	Hydrolysis of glycosidic bonds	PF03632,
Glycoside hydrolase family 72	51025 (438), 106219 (519)	Hydrolysis of glycosidic bonds	PF03198
Glycoside hydrolase family 75	96467 (219)	Hydrolysis of glycosidic bonds	PF07335
Glycoside hydrolase family 76	34014 (427), 45180 (392)	Hydrolysis of glycosidic bonds	PF03663
Glycoside hydrolase family 78	69329 (663)	Hydrolysis of glycosidic bonds	PF05592
Glycoside hydrolase family 92	35446 (748), 48260 (771), 74711 (767), 101742 (789)	Hydrolysis of glycosidic bonds	PF07971
Pectate lyase	85457 (306)	Cleavage of pectate	PF00544

PFAM information obtained from http://pfam.sanger.ac.uk/.

**Table 3:** The sub-set of *M. graminicola* genes that code for secreted proteins involved in the degradation of proteins.

Annotation	#id-JGI (amino acids)	Functional annotation	PFAM
Aspartic proteases	92644 (437), 92645 (466), 94263 (441), 107454 (462), 110047 (424), 110888 (514),	Hydrolysis of peptide bonds	PF00026
Peptidase_S8/serine proteases	34453 (376), 72659 (384), 91795 (256), 109122 (342), 70312 (492),	Hydrolysis of peptide bonds	PF00082
Zinc carboxypeptidase	59604 (403)	Hydrolysis of peptide bonds	PF00246
Peptidase_S10/Serine carboxypeptidase	68068 (554), 74336 (639), 75070 (489), 77689 (552), 90471 (601), 99840 (622), 103135 (534), 106874 (526), 109759 (527),	Hydrolysis of peptide bonds	PF00450
Peptidase family M3	38371 (725)	Hydrolysis of peptide bonds in medium sized peptides	PF01432
Peptidase_A4	90046 (231), 91855 (235), 105030 (251)	Hydrolysis of peptide bonds	PF01828
Deuterolysin metalloprotease (M35) family	39241 (350)	Hydrolysis of peptide bonds	PF02102
PA domain/Transferrin receptor-like dimerisation domain/Peptidase family M28	95621 (637)	Hydrolysis of peptide bonds	PF02225, PF04253, PF04389
PA domain/Peptidase family M28	65261 (534)	Hydrolysis of peptide bonds	PF02225, PF04389
Peptidase family S51	49854 (280)	Hydrolysis of peptide bonds	PF03575
Peptidase_M43	76021 (262)	Hydrolysis of peptide bonds	PF05572
Serine carboxypeptidase S28	66250 (540), 70079 (510), 76675 (512), 108506 (527)	Hydrolysis of peptide bonds	PF05577
Pro-kumamolisin, activation domain	37389 (603), 72506 (633), 75846 (591), 83794 (577), 84465 (640)	Hydrolysis of peptide bonds	PF09286

PFAM numbers and annotation were obtained from http://pfam.sanger.ac.uk/.

**Table 4:** The sub-set of *M. graminicola* genes that code for secreted proteins involved in the lipids degradation.

Annotation	#id-JGI (amino acids)	Functional annotation	PFAM
Pectinesterase	66866 (314)	Catalysis of the pectin de-esterification	PF01095
Coesterase/Carboxylesterase family	50790 (518), 72632 (561), 81448 (76*), 108908 (554), 110417 (461), 44636 (490), 72841 (476), 74078 (692), 75146 (514), 90758 (542)	Hydrolysis of carboxylic ester	PF00135
Esterase PHB depolymerase	95636 (315), 106075 (283)	Hydrolysis, hydrolysis of carboxylic ester	PF10503
Glycerophosphoryl diester phosphodiesterase family	40275 (395)	Hydrolysis of carboxylic ester	PF03009
Sulfatase	40096 (557), 76800 (583)	Hydrolysis of sulfate esters	PF00884
Tannase and feruloyl esterase	46238 (521), 49510 (552), 51439 (516)	Hydrolysis of carboxylic ester in digallic acid/digallate, a polyphenolic compound	PF07519
Phosphoesterase family	67329 (393), 72002 (413)	Hydrolysis of phosphodiester bond	PF04185
Cutinase	43394 (213), 68483 (197), 77282 (214), 99331 (206)	Hydrolysis of cutin carboxylic ester bonds	PF01083
Group XII secretory phospholipase A2 precursor	90411 (288), 96437 (292)	Hydrolysis, hydrolysis of carboxylic ester	PF06951
Lysophospholipase	64715 (558), 107391 (643)	Hydrolysis of 2-lysophosphatidylcholine	PF01735

PFAM numbers and annotation were obtained from http://pfam.sanger.ac.uk/.

(*) Appears to be an erroneous gene model.

**Table 5:** The sub-set of total putative secreted proteins related to plant polysaccharides degradation found in *M. graminicola*.

Substrate	Protein Name	Code	CAZy family	Number of copies^a^
Cellulose/Hemicellulose	β-1,4-glucosidase	BGL	**GH3**, **GH1**	8
	β-1,4-endoglucanase	EGL	**GH5**, **GH7**, **GH12**, **GH45**, **GH61** *	6
	Cellobiohydrolase	CBH	**GH6**, **GH7**	1
Galactomannan	β-1,4-galactosidase**	LAC	**GH2**, **GH35**	1
	β-1,4-mannosidase	MND	**GH2**	0
	α-1,4-galactosidase***	AGL	GH27, **GH36**	0
	β-1,4-endomannanase	MAN	**GH5**, GH26	0
Inulin	Inulinase	INU	GH32	1
Pectin	Feruloyl esterase	FAE	**CE1**	3
	Rhamnogalacturonan acetyl esterase	RGAE	CE12	0
	Pectin methyl esterase	PME	**CE8**	1
	Unsat.-rhamnogalacturonan hydrolase	URH	**GH105**	0
	β-1,4-galactosidase**	LAC	**GH2**, **GH35**	0
	Endo-/exo-(rhamno) galacturonase	-	**GH28**	1
	β-1,4-xylosidase****	BXL	**GH3**, **GH43**	5
	Endoarabinanase	ABN	**GH43**	0
	α-arabinofuranosidase	ABF	**GH51**, **GH54**	0
	β-1,4-endogalactanase	GAL	**GH53**	1
	α-rhamnosidase	RHA	**GH78**	1
	Unsaturated glucuronyl hydrolase	UGH	**GH88**	0
	Exoarabinanase	ABX	**GH93**	0
	Pectin lyase	PEL	**PL1**	0
	Pectate lyase	PLY	**PL1**, **PL3**, PL8	1
	Rhamnogalacturonan lyase	RGL	**PL4**, **PL11**	0
Starch	α-amylase	AMY	GH13	2
	Glucoamylase	GLA	GH15	1
Xylan	α-1,4-glucosidase	AGD	GH31	2
	Acetyl xylan/feruloyl esterase	AXE/FAE	**CE1**	2
	β-1,4-endoxylanase	XLN	**GH10**, **GH11**	2
	β-1,4-galactosidase**	LAC	**GH2**, **GH35**	0
	α-1,4-galactosidase***	AGL	GH27, **GH36**	1
	β-1,4-xylosidase****	BXL	**GH3**, **GH43**	0
	α-arabinofuranosidase	ABF	**GH51**, **GH54**	3
	Arabinoxylan Arabinofuranohydrolase	AXH	**GH62**	1
	α-glucuronidase	AGU	**GH67**, GH115	0
Xyloglucan	Xyloglucan β-1,4-endoglucanase	XEG	**GH12**, **GH74**	0
	β-1,4-galactosidase**	LAC	**GH2**, **GH35**	0
	α-1,4-galactosidase***	AGL	GH27, **GH36**	0
	α-fucosidase	AFC	**GH29**, **GH95**	0
	α-xylosidase	AXL	GH31	0
	α-arabinofuranosidase	ABF	**GH51**, **GH54**	0

(*) According to CAZy classification, GH61 enzymes are certainly non-canonical and they cannot be considered as bona fide glycosidases,

(**) Enzyme that could act on Pectin, Galactomannan, Xylan or Xyloglucan,

(***) Enzyme that could act on Galactomannan, Xylan or Xyloglucan,

(****) Enzyme that could act on Pectin or Xylan. In bold, enzymes that are related to plant cell wall degradation.

a – See Tabs 7 and 8 in File S1 for protein Ids.

**In bold**, enzymes that are related to plant cell wall degradation.

### Hydrophobin-like proteins

Fungal secreted hydrophobins facilitate attachment to hydrophobic surfaces. Typically, they have eight cysteines in the mature protein sequence [28]. In the original annotation of the *M. graminicola* genome eight hydrophobin-like proteins were predicted [12]. Of these 4 are found in both the total and the refined secretome (48129, 96543, 108349 and 88691), 2 were only identified in the total secretome (95491and 96536) whilst the other 2 proteins were not predicted to be secreted (40724 and 117719). The sizes of the 4 hydrophobin-like sequences predicted in the refined secretome are very different (96, 143, 463 and 816 aa's) and two contain a different number of cysteine residues (8, 8, 15 and 46, respectively). This finding suggests that the current gene models should be reassessed for at least two of the predicted secreted hydrophobins.

### The relative abundance of individual PFAM domains in the refined secretome

The refined *M. graminicola* secretome of 492 proteins contains 235 with at least one PFAM domain identified (see Tab 5 in File S1). Their relative abundance was determined and the most frequent PFAM domains identified (Table 6). The most frequently observed PFAM in the *M. graminicola* refined secretome was PF01328 corresponding to peroxidase_2. This was detected in 11 predicted secreted proteins. Other PFAMs present in high copy numbers included PF00135 corresponding to carboxylesterase present in 10 proteins, PF00732 corresponding to the glucose-methanol-choline oxidoreductase family (FAD ADP-binding domain) present in 9 proteins, PF00450 corresponding to Peptidase_S10 is present in 9 proteins and PF05199 corresponding to GMC_oxred_C (steroid-binding domain) is present in 8 proteins. Two *M. graminicola* proteins in the refined secretome possessed a very large number of PFAM domains. These were protein Id 95631 (PF02993, PF03154, PF03276, PF04652, PF05109, PF05518, PF06070, PF07174, PF08639, PF09726, PF09770, PF10667). However there is no clear function associated with these and manual inspection suggested the protein to be very rich in serine, threonine and proline residues. Protein Id 109621 possessed 5 predicted PFAM motifs (PF04625, PF06676, PF07174, PF10287 and PF10290) and was also rich in these particular amino acids but with no clear function predicted.

**Table 6:** Most frequent PFAM domains found throughout the secretome of *Mycosphaerella graminicola* (*Mg*), and corresponding frequency in *Fusarium graminearum* (*Fg*).

PFAM	*Mg*	*Fg*	Description
PF01328	11	2	Peroxidase_2
PF00135	10	14	Carboxylesterase
PF00732	9	8	Glucose-methanol-choline oxidoreductase family (FAD ADP-binding domain)
PF00450	9	7	Peptidase_S10
PF05199	8	7	GMC_oxred_C (steroid-binding domain)
PF00933	7	8	Glyco_hydro_3
PF01915	7	8	Glyco_hydro_3_C
PF00264	6	7	Tyrosinase
PF07859	6	8	Abhydrolase_3
PF00026	6	6	ASP Eukaryotic aspartyl protease
PF09286	5	2	Pro-kuma_activ
PF01565	5	16	FAD_binding_4
PF00082	5	12	Peptidase_S8
PF08760	4	0	DUF1793
PF07971	4	0	Glyco_hydro_92
PF05577	4	1	Peptidase_S28
PF01593	4	1	Amino_oxidase
PF01083	4	9	Cutinase
PF00722	4	8	Glyco_hydro_16

### Analysis of secreted proteins with no predicted enzymatic functions

A total of 171 proteins present within the refined *M. graminicola* secretome are currently described as hypothetical or conserved hypothetical proteins (Tab 6 in File S1). We therefore explored these sequences for the presence of a number of interesting features.

#### a. Tandem repeat containing proteins

We have previously described a small family of predicted secreted proteins possessing internal tandem coding repeats, referred to as the MgTRPs (*M. graminicola* Tandem Repeat Proteins), some of which showed increased expression during plant infection [29]. The original prediction for secretion of these twenty-three proteins arose from SignalP scores and WoLF PSORT predicting extracellular as the most likely protein location. The current analysis predicted 21 of these proteins in the total secretome but did not predict secretion for MgTRP4 or MgTRP17 (Tab 1 in File S1). In contrast the refined analysis predicted secretion for only MgTRP1, 2, 14, 15, 16 and 17 (Tab 4 in File S1).

#### b. Putative effector and cysteine-rich secreted proteins

*M. graminicola* isolate IPO323 possesses sequence homologues of a number of functionally characterised effector proteins identified in the tomato leaf mould pathogen *Cladosporium fulvum*. These include two *in planta* expressed homologues of CfECP6, referred to as Mg3LysM and Mg1LysM, which have demonstrated effector functions in *M. graminicola* with both similar and unique properties [16]. *M. graminicola* also has three putative homologues of the *C. fulvum* effector ECP2 which are referred to as MgECP2, MgECP2-1 and MgECP2-2 [30]. Finally *M. graminicola* possesses one functional copy of the secreted necrosis and ethylene inducing proteins (NEPs) referred to as MgNLP. This protein does not play a significant role in virulence but its transcript is strongly up-regulated during leaf infection [24]. Our analysis of both the total and refined predicted secretome of *M. graminicola* identified all of these predicted proteins in both categories (Tabs 1 and 4 in File S1) adding further weight to our predictive approach. By analysing proteins containing 6 or more cysteines in the refined secretome, we identified 94 proteins (Tab 10 in File S1).

In fungi and oomycete plant pathogens a number of small (<200 amino acids) cysteine-rich apoplastic effectors have been described which lack homology to proteins in other species. We therefore examined the refined *M. graminicola* secretome for the presence of putative effectors of this type. In the refined secretome an overall total of 70 proteins have cysteine representing over 5% of the mature protein length. Intriguingly for all but 9 of these predicted proteins the mature protein length was less than 200 amino acids with a mean overall length of 143 amino acids. Eight of the 9 cys-rich proteins larger than 200 amino acids have some form of functional annotation. In contrast, of the 61 cys-rich proteins below 200 amino acids in length only 11 have any functional annotation. Therefore a total of 50 proteins have >5% cysteine and no functional annotation (Table 7) with a smaller sub-set of 22 proteins being comprised of more than 10% cysteine (Tabs 11 and 12 in File S1). As anticipated there were no putative cys-rich proteins detected on any of the dispensable chromosomes 14–21 and the largest number on any one chromosome was detected on chromosome 13 (seven proteins – Table 7).

**Table 7:** The predicted <200 amino acid cysteine-rich proteins (>5% cys) from *M. graminicola* isolate IPO323.

#id-JGI	Chr	Pre len	Sig len	Mature len	Num cys	%C	Mg specific (<e-5)
87205	8	55	22	33	8	24.24	Y
83081	13	53	20	33	6	18.18	Y
104444	5	79	21	58	10	17.24	Y
82925	12	57	19	38	6	15.79	Y
81208	6	59	18	41	6	14.63	Y
82029	9	66	18	48	7	14.58	Y
101652	11	77	21	56	8	14.29	Y
79286	2	63	20	43	6	13.95	Y
100649	7	75	17	58	8	13.79	Y
41491	5	81	18	63	8	12.7	N
79161	1	68	19	49	6	12.24	Y
99161	2	164	15	149	18	12.08	N
106125	11	70	17	53	6	11.32	Y
106502	13	89	18	71	8	11.27	Y
108482	3	108	19	89	10	11.24	N
80332	4	76	21	55	6	10.91	Y
104383	5	74	19	55	6	10.91	Y
83064	13	75	18	57	6	10.53	Y
104758	6	118	22	96	10	10.42	Y
97031	12	118	22	96	10	10.42	Y
106445	13	119	22	97	10	10.31	Y
105826	10	98	20	78	8	10.26	Y
97077	12	104	19	85	8	9.41	N
95035	8	106	19	87	8	9.2	Y
97500	13	137	28	109	10	9.17	Y
89647	1	109	20	89	8	8.99	Y
92365	4	106	16	90	8	8.89	Y
96910	12	152	16	136	12	8.82	N
94383	7	112	21	91	8	8.79	Y
102617	1	158	20	138	12	8.7	Y
96536	11	165	16	149	12	8.05	N
99124	2	112	20	92	7	7.61	Y
96101	10	132	21	111	8	7.21	Y
95714	9	104	18	86	6	6.98	N
97449	13	105	19	86	6	6.98	Y
96389	10	109	20	89	6	6.74	Y
97526	13	205	25	180	12	6.67	N
108877	3	111	20	91	6	6.59	Y
107286	1	116	22	94	6	6.38	Y
88664	1	145	19	126	8	6.35	N
93609	6	188	20	168	10	5.95	Y
90533	2	138	19	119	7	5.88	N
88698	1	159	22	137	8	5.84	Y
93075	5	193	20	173	10	5.78	Y
102996	1	163	23	140	8	5.71	N
110386	8	194	17	177	10	5.65	N
110220	7	131	19	112	6	5.36	Y
96865	11	131	19	112	6	5.36	Y
95574	9	114	18	96	5	5.21	Y
94117	6	137	17	120	6	5	N

### Analysis of sequence motifs associated with fungal and oomycete effectors

All 492 refined secretome sequences were inspected for the presence of the degenerative RxLR-dEER [31] and Y/F/WxC motifs [32] located in close proximity to the predicted signal peptide sequence. No exact RxLR-dEER matches were found within the refined *M. graminicola* secretome. By contrast, the degenerative Y/F/WxC motif was present in close proximity to the predicted signal peptide cleavage site in 16 proteins (Tab 4 in File S1). All three motifs were identified YxC (5), FxC (5) and WxC (6) and 10 of these proteins had a mature length of <150 amino acids. Only three proteins were annotated, protein Id 37166 a xyloglucan xyloglucosyl transferase (WxC), protein Id 76589 a Glycosyl hydrolase family 45 member with the PFAM domain PF02015 (WxC) and protein Id 109621 with 5 PF domains (PF04625, PF06676, PF07174, PF10287, PF10290) (FxC). In addition, the Y/F/WxC motifs were found in the correct location in 10 hypothetical proteins with no annotation (Tab 6 in File S1).

Upon inspection of the refined secretome dataset for the presence of bipartite nuclear localisation signals (NLS), no proteins containing this motif were identified (Tab 4 in File S1). By contrast 130 proteins were predicted to contain at least one nuclear export sequence (NES) (Tab 4 in File S1).

### Interspecies comparative analyses

Initially, these comparative analyses on the predicted refined secretome were done between the cereal ear and stem base infecting ascomycete species *F. graminearum* and the solely wheat leaf infecting *M. graminicola.* This revealed there were dramatic differences in both the number of predicted PCWDEs as well as the repertoire of other PFAM domain present (Table 6). As stated previously, the most frequently observed PFAM in the *M. graminicola* refined secretome was PF01328 which corresponds to peroxidase_2, detected in 11 copies. Whereas in the refined *F. graminearum* (*Fg*) secretome only 2 copies of PF01328 were identified. This represents a major quantitative difference between the two species. Other PFAMs clearly identified more frequently in the *Mg* refined secretome include PF09286 (Pro-kuma_activ protease), PF08760 (DUF1793), PF07971 (Glyco_hydro_92 glycosylhydrolase), PF05577 (Peptidase_S28) and PF01593 (Amino_oxidase). Conversely a number of PFAMs frequently identified in the *Fg* refined secretome were less frequent or absent from the *Mg* refined secretome (Table 8). Notably several PFAMs encoding different glycosyl hydrolases were reduced in number including PF04616, PF03443, PF00722, PF00704 and PF00295. *Mg* also has less predicted secreted proteins possessing the PFAMs; PF01083 (Cutinase), PF00544 (Pec_lyase_C pectate lyase), PF00150 (Cellulase) and PF01522 (Polysacc_deac_1) which may be implicated in the degradation of plant cell walls (as alluded to in the previous section). In addition, several other PFAMs frequently observed in the *Fg* refined secretome were entirely absent from the *Mg* secretome, including PF00657 (Lipase_GDSL), PF08031 (BBE Berberine bridge-like enzymes) and PF09044 (Kp4 killer toxin). Therefore there are clear qualitative differences overall in the protein activities predicted to reside in the refined secretomes of the two tissue specific fungal pathogens of wheat.

**Table 8:** Most frequent PFAM domains found throughout the secretome of *Fusarium graminearum* (*Fg*), and corresponding frequency in *Mycosphaerella graminicola* (*Mg*).

PFAM	Fg	Mg	Description
PF01565	16	5	FAD_binding_4
PF00135	14	10	Carboxylesterase
PF00082	12	5	Peptidase_S8
PF04616	11	3	Glyco_hydro_43
PF01083	9	4	Cutinase
PF03443	9	2	Glyco_hydro_61
PF00657	9	0	Lipase_GDSL
PF00933	8	7	Glyco_hydro_3
PF01915	8	7	Glyco_hydro_3_C
PF00732	8	9	GMC_oxred_N
PF00722	8	4	Glyco_hydro_16
PF08031	8	0	BBE (berberine bridge- like enzymes)
PF07859	8	6	Abhydrolase_3
PF05199	7	8	GMC_oxred_C
PF01822	7	2	WSC domain
PF00544	7	1	Pec_lyase_C
PF00450	7	9	Peptidase_S10
PF00264	7	6	Tyrosinase
PF00150	6	2	Cellulase
PF00704	6	2	Glyco_hydro_18
PF05109	6	2	Herpes_BLLF1 (outer envelope glycoprotein)
PF00026	6	6	ASP Eukaryotic aspartyl protease
PF00295	5	1	Glyco_hydro_28
PF01522	4	0	Polysacc_deac_1
PF09044	4	0	Kp4 (Killer toxin)
PF00144	4	2	Beta-lactamase

**Figure 4:**
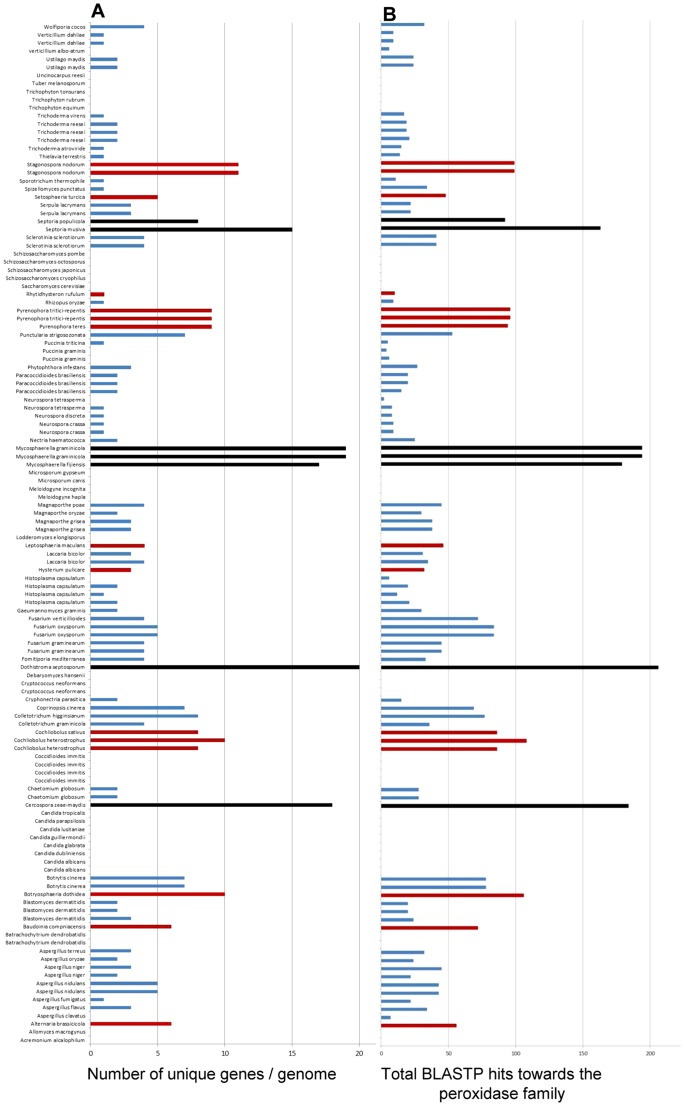
Interspecies genome BLASTP counts for homologues of the *M. graminicola* secreted protein family with peroxidase/chloroperoxidase activity (PFAM; PF01328). (A) The number of unique protein homologues of protein 90087, a representative member of the secreted peroxidase family. (B) Total number of BLASTP hits in each species towards the 11 predicted secreted peroxidases from *M. graminicola*. Black bars highlight *Mycosphaerellaceae* species; red bars highlight other Dothideomycetes. Analysis performed at a BLASTP cut-off of e-5.

The 492 members of the refined *M. graminicola* secretome were then subjected to an extensive interspecies comparison based upon 126 genome datasets representing 88 fungal species (including two genome databases for *M. graminicola* itself), 1 plant parasitic oomycete (*Phytophthora infestans*) and 2 plant parasitic nematodes (*Meloidogyne incognita* and *M. hapla*). The fungi analysed included predominantly ascomycete and basidiomycete species, one mucoromycotina and spanned a range of lifestyles including micro and macro saprophytes as well as plant and animal pathogens (Table S1). They include 17 additional Dothideomycete species, 14 of which are recognised as plant pathogens. Within this class were 5 additional members of the *Mycosphaerellaceae* (Tabs 13 and 14 in File S1). This analysis revealed a remarkable predicted expansion of homologues of the eleven predicted secreted peroxidases possessing PFAM domain PF01328 in the *Mycosphaerellaceae* and to a lesser extent to other members of the plant pathogenic Dothideomycetes (Figure 4). BLASTP analysis with one member of this putative protein family (protein Id 90087) identified a total of 19 unique proteins in the genome of *M. graminicola* (Tab 18 in File S1 and Figure 4A). Similarly high numbers were found in the genomes of the *Mycosphaerellaceae* species, including *Cercospora zeae-maydis* (18), *Dothistroma septosporum* (20), *M. fijiensis* (17) and *Septoria musiva* (15), whilst slightly fewer were observed in *Septoria populicola* (7). This latter number was similar to other non- *Mycosphaerellaceae* plant pathogenic Dothideomycetes including *Stagonospora nodorum* (12) which possessed the most homologues outside the *Mycosphaerellaceae* species, and *Pyrenophora tritici-repentis* and *P. teres* (9 each). The Dothideomycetes with the fewest homologs were *Rhytidhysteron rufulum* which colonises wood or other dead plant tissues (1) and the saprophyte *Hysterium pulicare* (3). Outside the Dothideomycetes most homologues were found in the plant pathogen *Colletotrichum higginsianum* (8) whilst *Fusarium graminearum* was predicted to have four homologues. The overall pattern described for protein 90087 was also observed when total BLASTP hits from all 11 members of the predicted *M. graminicola* secreted peroxidases were plotted (Figure 4B). Homologues of these genes were notably absent from all analyses members of the *Saccharomycotina* including the animal pathogens (*Candida* species) and the free living yeasts (*Saccharomyces* and *Schizosacharomyces* species).

#### a. The distribution of M. graminicola functionally uncharacterised cysteine rich proteins

The global comparative analysis of all 492 proteins from the refined secretome against these 126 genome datasets also identified 85 proteins that may be unique to *M. graminicola* (no homologues identified in any species at an e value cutoff of <10^−5^) (Tabs 15–18 in File S1). None of these 85 proteins were located on the dispensable chromosomes (Figure 2C). Of the 85 potentially *M. graminicola* specific proteins, 38 (45%) were cysteine-rich (>5% cys) (Tables 1 and 7). Some predicted secreted proteins also had homology to one another (i.e. they could be considered twins) or to genes present in the genome sequence currently not predicted to encode secreted proteins (Table 9). Amongst, these predicted 85 unique *M. graminicola* proteins, 10 possessed the Y/F/WxC motifs [32] located in close proximity to the predicted signal peptide sequence (Tab 17 in File S1). Of these, 5 proteins had a cysteine content >10%.

**Table 9:** Features of the predicted secreted proteins unique to *M. graminicola* based on interspecies BLASTP analysis (e-5).

#id-JGI	Chr	gpi	Pre len	Sig len	mature len	numcs	%Cys	No. of homologues in Mg genome (<e-5)
**104444**	5	-	79	21	58	10	17.24	1
**104758** **	6	-	118	22	96	10	10.42	1**
**106445** **	13	-	119	22	97	10	10.31	1**
**105826** ***	10	-	98	20	78	8	10.26	1***
**89647** ***	1	-	109	20	89	8	8.99	1***
**102617**	1	-	158	20	138	12	8.7	1
**94557**	7	-	365	20	345	25	7.25	1
**96389**	10	-	109	20	89	6	6.74	1
**93609** *	6	-	188	20	168	10	5.95	2*
**93075** *	5	-	193	20	173	10	5.78	2*
**96865**	11	-	131	19	112	6	5.36	1
**96869**	12	-	183	18	165	6	3.64	1
**110633** ****	8	Y	357	15	342	9	2.63	3****
**110142**	7	-	318	19	299	6	2.01	1
**105608** ****	8	Y	503	15	488	8	1.64	3****
**92880**	4	-	369	26	343	3	0.87	1
**104754**	6	-	163	16	147	0	0	1

*/**/***/**** highlight proteins with homology to one another in the refined secretome.

In total, 12 functionally unclassified cys-rich proteins possessed homologues amongst the other species analysed. The distribution of these predicted proteins varied across the genomes analysed. For example 11 of these proteins had a homologue in the genome sequence of at least one other Dothideomycete species (Tab 15 in File S1). In almost every case at least one copy was found in the related maize leaf infecting species *Cercospora zeae-maydis*. Homologues of one cys-rich protein in particular (97526) were found only in this related species, appearing as a small gene family comprising six members. Whilst, homologues of protein Id 102996, which is both cysteine rich and possess a YSC motif nearby the signal peptide, was found only in *Cercospora zeae-maydis* as a single copy gene. The distribution of homologues of the other conserved proteins varied considerably. Whilst many were detected only in related *Mycosphaerellaceae* pathogens (eg proteins 95714, 96536, 97077, 97526, 88664, 90533 and 108482) others had more widespread distribution. Homologues of protein 41491 were identified in fungal pathogens and saprophytes but curiously not from any other *Mycosphaerellaceae* species or any other Dothideomycete fungus. Homologues of protein 96910 were only found in plant pathogenic fungi whilst homologues of protein 110386 were most widespread overall indicated by their presence in 71 of the queried genome databases representing over 50 different fungal species (Tab 15 in File S1).

#### b. Copy number and distribution of the functionally non-annotated sequences

The 171 predicted secreted proteins with no annotation were inspected for copy number in *M. graminicola* itself, and in 90 other species of predominantly fungi, but also including the oomycete *P. infestans* and the nematodes *M. incognita* and *M. hapla* (Tab 15 in File S1). To do this each protein was subjected to BLASTP analysis against each of the named genomes using a cut off value of e-5 and the unique protein hits then identified. Sixty-three predicted proteins were found to be *M. graminicola* species specific, of which 48 were single copy, 11 were present in two copies and 4 as three copies. For the sequences 91702, 90420, 41491 and 107811 although no homologous sequences were found in other Dothideomycete species, similar sequences were found in one or more species outside this class (Tab 19 in File S1). This rare distribution suggests these novel sequences may have arisen from elsewhere in the fungal kingdom.

The distribution of some of the *M. graminicola* sequences within the 17 Dothideomycete species was particularly striking (Table 10). For example, protein 91252 was found as 11 copies in *M. graminicola*, but was present between 0 and 4 copies in the taxonomically related species. The unrelated proteins 68660, 89162 and 89375 were found as 7, 6 or 4 copies in *M. graminicola*, but were present between 0 and 14 copies, 1 and 8 copies and 0 to 8 copies, respectively, in the other species. The related proteins 32157 and 33493 were found in 5 copies in *M. graminicola* but between 4 and 13 copies in the other Dothideomycete. A number of the other sequences were particularly prevalent in a single species. For example, the related proteins 107050 and 73873 were most prevalent in *Botryosphaeria dothidea*, 6 or 7 copies versus 4 in *M. graminicola.* Whilst, 77324 present only as a single copy in *M. graminicola*, had 12 copies in *Botryosphaeria dothidea* and 97526 again a single copy sequencing in *M. graminicola* had 6 copies in *Cercospora zeae –maydis* and none in the other Dothideomycete species. The species distribution of a few of the single copy *M. graminicola* proteins was also particularly striking. For example, 110386 was present between 12 and 17 copies in the two *Cochliobolus* species whereas the other Dothideomycete species had only a few copies (<6 throughout). In contrast, it was noticeable that for a few proteins, the copy number was slightly higher in *M. graminicola* compared to any of the other Dothideomycete species. Finally, 15 proteins had a very narrow taxon distribution being found only in *M. graminicola* and one other Dothideomycete species.

**Table 10:** Distribution and copy number of *M. graminicola* homologues in other *Dothideomycetes* and other fungal species.

Gene copy number	Total No.	Genes with no annotation	*Mg* specific	Gene not in another Doth* species, but found in other fungal species (name abbreviation)	Genes with large difference in copy number per Doth species (name abbreviation)	Low numbers or no copy in other Doth species	Only found in one other Doth species (name abbreviation)
**10 and above**	68	1	0	0	0	**91252**	0
**6–9**	41	3	0	0	**68660** (Bc, Ds, Mf), 108321, **89162**	0	0
**5**	25	2	0	0	**32157**, **33493** (Ch, Cs, St)	0	0
**4**	32	11	0	0	92962, 95631, 105677, 105896, **107050** (Bc), 44587, 72728 (Lm), **73873** (Bc), **89375** (Ds), 93903, 96426,	104794 111027	0
**3**	50	13	4	0	**108329**, **41315**, 43397	91995 92156 92790 95537 96868 96876 99676	89705 (Mf) 96876 (Cmz) 95537 (Ds)
**2**	72	29	11	91702 (Tv)	103460 (Ds), 103564 (Bc, Ds), 105223, 33309 (St), 67799, 71681 (Lm), 75316 (Sm), 88916 (Ch), 95831	104444 106021 106106 95714 95788 96536 96910	90533 (Czm)
**1**	204	112	48	107811 (Cg, Tt, Vd, Va-r, Vd), **41491** ** (13 species^a^), **90420** (15 species^a^), 97283 (Tv)	108976, **110386** (several), 111505, 29006 (Bc), 34196, 65051, **68477**, 70376, **77324** (Bc, Rr), 91361, 92097, 93838, 94408, 95672, **97526** (Czm), 99161, 88664, 94117, 99350	89079 89878 90699 92048 92094 92990 92998 94017 94299 94840 95108 97077 96943	102996 (Czm) 104000 (Lm) 108482(Mf) 73448 (Cs) 82659 (Ds) 90503 (Bc) 90532 (Bc) 91471 (Mf)
**TOTAL**	**492**	**171**	**63**	**5**	**47**	**30**	**12**

BLASTP analysis e^−5^.

* Doth – *Dothideomycete* species;

** Protein ID in bold, indicates additional information in main text;

a – see Tabs 15 and 18 in File S1 for full species list distributions.

#### c. Global analysis of all refined secreted proteins shared with other organisms

Of the 407 *M. graminicola* predicted proteins which had homologues in at least one other species analysed, the largest number of homologues were found in the maize leaf infecting *Mycosphaerellaceae* species *Cercospora zeae-maydis* with 352 of the 407 having at least one homologue at e-5. Tabs 15 and 18 in File S1 highlight that there was an overall trend for the largest number of homologues to reside in the predicted proteomes of other *Mycosphaerellaceae* species. These were then followed by other Dothideomycete cereal infecting pathogens and then other plant pathogenic fungi (Table 11). The organisms that had fewest homologues to the 407 *M. graminicola* proteins were the plant parasitic nematodes (only 55 and 56 of the 407 possessed homologues respectively) followed by the ascomycete budding yeasts (typically having <112 with homology to the 407), the plant parasitic oomycete *P. infestans* (125) then the cereal infecting basidiomycete *Puccinia* species (<134) and finally lower fungi from the phylum Chytrids (<144).

**Table 11:** Conservation of the *M. graminicola* (*Mg*) genes, predicted to encode secreted proteins, amongst the 124 fungal, oomycete and Chytrid genomes assessed and then presented according to overall species distribution or lifestyle.

	Refined secretome	Total secretome
Total number of genes	492	970
*Mg* specific	85	234
All Dothideomycetes species	54	118
All Dothideomycetes pathogenic species	42	90
All Ascomycete species	132	262
Ascomycete pathogens	63	109
Ascomycete plant pathogen	60	99
Ascomycete animal pathogen	3	5
Ascomycete saprophyte	0	5
All plant pathogens	64	112

Probability value e-5.

This analysis revealed nine proteins from the *Mycosphaerella graminicola* secretome only found in fungal pathogens of wheat plants or other cereals (Table 12), including an expansion with six copies of the 97526, a protein with unknown function, in the genome of *Cercospora zeae-maydis*, a fungus of the class Dothiodiomycete that causes the gray leaf spot, a foliar disease of corn, and an expansion of the hydrophobin-like protein 92962, with five other homologues in the genome of *M. graminicola* (108349, 109435, 95631, 94883 and 96944), all secreted. In addition, the gene 92805, with PFAM domain related to fibronectin attachment, shows a unique homologue in *Fusarium verticillioides*, a very common fungal species (class Ascomycete) that can infect all organs of maize plants. However, only the 102996, a protein without function assigned, found exclusively in *C. zeae-maydis* shows EST support so far (Tab 20 in File S1).

**Table 12:** Number of putative secreted proteins from *Mycosphaerella graminicola* secretome only found in fungal pathogens of wheat plants or other cereals.

#id-JGI	Wheat host											Other cereal hosts						EST	No. of copies in Mg	Annotation
	BO	CC	CO	CP	CR	DK	DM	BN	DO	DP	EG	BE	BL	EE	CE	CQ	EQ			
102996	-	-	-	-	-	-	-	**-**	**-**	**-**	**-**	**1**	**-**	**-**	**-**	-	-	Y	1	Unknown
88665	-	-	-	-	-	-	-	**-**	**-**	**-**	**-**	**2**	**-**	**-**	**-**	-	-	N	1	Unknown
96876	-	-	-	-	-	-	-	**-**	**-**	**-**	**-**	**1**	**-**	**-**	**-**	-	-	N	1	Unknown
97526	-	-	-	-	-	-	-	**-**	**-**	**-**	**-**	**6**	**-**	**-**	**-**	-	-	N	1	Unknown
90533	-	-	-	-	-	-	-	**-**	**-**	**-**	**-**	**1**	**-**	**-**	**-**	-	-	N	2	Unknown
92747	-	-	-	-	-	-	-	**-**	**-**	**-**	**-**	**1**	**-**	**-**	**-**	-	-	N	1	Exo-alpha-sialidase
92805	-	-	-	-	-	-	-	**-**	**-**	**-**	**-**	**-**	**-**	**-**	1	-	-	N	1	Fibronectin-attachment protein
92962	-	-	-	-	-	-	-	**-**	**-**	**-**	**-**	**1**	**-**	**-**	**-**	-	-	N	6	Hydrophobin-like protein
94077	-	-	-	-	-	-	-	**-**	**-**	**-**	**-**	**1**	**-**	**-**	**-**	-	-	N	4	Unknown

BLASTP cut-off of e-5.

BO-*Colletotrichum graminicola*, CC-*Fusarium graminearum*, CO-*Gaeumannomyces graminis*, CP-*Magnaporthe oryzae*, CR-*Magnaporthe grisea*, DK-*Puccinia graminis*, DM-*Puccinia triticina*, BN-*Cochliobolus sativus*, DO-*Pyrenophora teres*, DP-*Pyrenophora tritici-repentis*, EG-*Stagonospora nodorum*, BE-*Cercospora zeae-maydis*, BL-*Cochliobolus heterostrophus*, EE-*Setosphaeria turcica*, CE-*Fusarium verticillioides*, CQ-*Magnaporthe poae*, EQ-*Ustilago maydis*. In File S1 is given the host species distribution of each pathogen.

**In bold** – Dothideomycetes.

For completeness, we included the BLASTP analyses results with e-values less than e-40 and e-100 in the Tabs 21 and 22 in File S1 and the analysis applied to secreted proteins found in other plant pathogenic fungi in the Tab 23 in File S1 and Table S2 for comparison.

### EST analysis of the predicted *M. graminicola* unique genes

To provide some further annotation for the 85 *M. graminicola* unique genes, and also to explore for any possible biological roles these may genes play, their presence/absence was explored in the previously published ESTs libraries prepared from the 9 *in vitro* conditions and 4 *in planta* conditions [22], [23]. Interestingly, 9 sequences were only present in one or more of the *in planta* derived libraries, 6 were present in at least one *in vitro* and one *in planta* library, whilst a further 24 were only present in one or more of the *in vitro* libraries. The three *M. graminicola* unique genes with the highest level of EST support were 80321 and 99917, present in most of the *in vitro* and *in planta* libraries and 105608 present in the most of the *in vitro* libraries. For completeness, the EST support present in the 13 libraries for each of the 85 *M. graminicola* unique genes as well as the rest of the refined secretome gene set is provided (Tab 20 in File S1). In total, 39 of the 85 *M. graminicola* unique genes had EST support.

## Discussion

Septoria tritici blotch disease of wheat caused by *Mycosphaerella graminicola* represents a significant economic threat to global wheat production in the context of future food security concerns. It is also emerging as another model pathosystem to investigate mechanisms of fungal pathogenesis in plants and to a lesser extent host resistance responses [15]. Re-sequenced genomes of specific isolates and new molecular tools are fast appearing to investigate important questions relating to host specificity and mechanisms of infection [8]. This latter point is clearly of interest as this would appear to differ in one clear respect to that used by the more established models, in particular *Magnaporthe oryzae* and *Fusarium graminearum*, in that the entire cereal infection process occurs without host cell penetration. This suggests that the *M. graminicola* fungus must deploy an intricate means of communication with plant cells from the extracellular (apoplastic or intercellular) environment to facilitate infection. It is likely that this fungus uses various secreted protein effectors to assist the leaf infection process. Moreover this mode of plant infection would appear to be widespread amongst the *Mycosphaerellaceae* pathogens in the fungal class Dothideomycetes. These pathogens collectively are responsible for widespread and devastating diseases of major cereal and non-cereal crop plants as well as several cultivated tree species [33].

The current study has made use of the finished genome of what is regarded as the reference isolate of *M. graminicola*, IPO323. This isolate possesses 21 chromosomes, currently the largest number identified for any individual strain. However, the smallest eight chromosomes are dispensable for plant infection [12], [13]. We aimed to provide here both a “total” and “refined” predicted protein secretome for this fungus. The reason for this is the current lack of experimental proteome data for this fungus in which to verify our predictions. Therefore we chose to predict those that have some likelihood of secretion (total secretome) along with those with even greater likelihood (refined secretome) on the basis of the available bio-informatic resources. It was recently determined that *M. graminicola* has functional homologs of ECP6, a chitin binding secreted protein effector from the *Mycosphaerellaceae* species *Cladosporium fulvum*, which infects the leaves of the dicotyledonous tomato plant [16]–[18]. The analysis presented here identified both *M. graminicola* LysM effectors within the refined predicted secretome adding further weight to our approach. Moreover these data highlight the power of effector discovery by comparative genomics. However many secreted protein effectors identified in *C. fulvum* or various other plant pathogenic fungi and oomycetes are unique to these species, which complicates the identification of other key effector proteins. For this reason we performed a genome wide *in silico* analysis of the predicted *M. graminicola* proteome and compared this to fungi and oomycetes for which predicted proteome data are available to explore further this secretome. The set of 492 secreted proteins represents 4.4% of the *M. graminicola* genome, which is in accordance with many other predicted secretomes [34].

Global analyses highlighted several interesting features of the refined *M. graminicola* secretome. Firstly no members were predicted to reside on the eight smallest dispensable chromosomes perhaps supporting their redundant (or at least currently cryptic) roles in plant infection. Secondly we found no evidence for specific micro-regions or clusters of secreted proteins in contrast to what has been discovered for effector proteins in the genome of the basidiomycete plant pathogen *Ustilago maydis* [35]. Thirdly there was no particular association of the distribution of the 492 predicted secreted proteins with regions of the genome containing repetitive DNA or transposable elements (data not shown). This distinguishes *M. graminicola* from other Dothideomycete plant pathogens including *Leptosphaeria maculans* which possesses a variety of effector proteins in such regions [36]. Fourthly the global interspecies analysis identified that most homologues of the 492 proteins were found in the genome of the related *Mycosphaerellaceae* species *Cercospora zeae-maydis* which like *M. graminicola* is a leaf-specific cereal pathogen, but in this case of maize. The next most similar set of species were all plant pathogenic species of the *Mycosphaerellaceae* including *Dothistroma septosporum* a foliar pathogen of many pine species; *Mycosphaerella fijiensis* a foliar pathogen of banana causing Black Sigatoka disease and *Septoria musiva* and *S. populicola* which cause leaf spots and cankers on poplars, the latter most commonly affecting poplars of the section Tacamahaca and theirs hybrids. The ‘most closely related’ list then generally moves into other members of the Dothideomycetes with the exception of the presence of *Colletotrichum graminicola*, a plant pathogenic member of the Sordariomycetes. Interestingly this fungus is considered an archetypal hemibiotroph [37] and also displays specificity to leaves of graminaceous plants thereby suggesting some similarities in mode of infection and host specificity to *M. graminicola.* At the other end of the spectrum the low similarity to the ascomycete budding yeasts (members of the Saccharomycotina) is notable, perhaps highlighting specialisations associated with filamentous growth and/or plant pathogenesis.

The composition of the 492 proteins in the refined secretome is also noteworthy. In total, 65% had some annotation leaving 35% lacking annotation. Amongst the annotated genes were a number of secreted glycoside hydrolases implicated in plant cell wall degradation. However this number was comparatively small when compared for example with the number reported in the predicted refined secretome of the cereal ear, stem base and root infecting fungus *Fusarium graminearum* [21] (and this study) (Figure 3). Whilst there is evidence for initial intercellular colonisation of wheat ears by *F. graminearum* prior to cell wall attack [38], this difference in numbers most likely reflects the reduced capacity of the strictly apoplastic colonising non cell penetrating hyphae of *M. graminicola* to breach the cell walls of living plant cells, as had been previously suggested from analysis of the total genome content [12]. The analysis of PFAM domain abundance between the two secretomes of these cereal attacking fungi was also particularly striking and revealed many differences (Tables 6 and 8). The most abundant PFAM in the *M. graminicola* secretome was PF01328 corresponding to peroxidase_2 or “chloroperoxidase”. This was present in 11 members of the refined secretome. In contrast this was found in only two members of the *F. graminearum* refined secretome. More remarkably the global interspecies BLASTP analysis clearly highlighted that almost all of the analysed *Mycosphaerellaceae* plant pathogens contained similarly high numbers of these predicted proteins, above and beyond the numbers found in other Dothideomycete plant pathogens, and non-Dothideomycete fungi (plant pathogens, animal pathogens or saprophytes). Analysis of the physical distribution of these genes in the *M. graminicola* genome did not suggest clustering (Figure S1). These predicted secreted proteins function in various processes ranging from halogenation of natural products (eg synthesis of the antibiotic chloramphenicol) but also perhaps significantly all use H_2_O_2_ as a substrate [39]. Hydrogen peroxide and related reactive oxygen species (ROS) are well studied components of inducible plant defence responses [40]. With respect to *M. graminicola* infection of wheat leaves roles for H_2_O_2_ in inhibiting (or slowing) initial colonisation by the fungus have been suggested [41]. H_2_O_2_ is also produced in very large amounts during disease lesion formation and asexual sporulation [10], [42], [43]. It is therefore tempting to speculate that this enlarged family of putative secreted chloroperoxidases might in part allow the fungus to overcome initial plant defences and/or allow hyphae to tolerate environments with high H_2_O_2_ and oxidative stress typical of photosynthetically active leaves. The high numbers of predicted protein homologues in most of the plant pathogenic *Mycosphaerellaceae* species we studied, suggests that this may be an adaptation important for plant infection by many members of this family of fungi. This attribute might distinguish them from other plant pathogenic fungi with different tissue specificities and/or modes of infection.

Based on the interspecies BLASTP analysis a total of 85 predicted secreted proteins appeared to be unique to *M. graminicola* and most had no annotation. A significant proportion of these (37) were deemed to be small (<200 aa's) and cysteine-rich, which are features particularly well described for apoplastic effector proteins identified from a number of fungi and oomycetes [44], [45] . Effector proteins are considered to be virulence factors which assist colonisation by the pathogen through interfering with the activation of plant defence responses or counteracting components of these defence responses. They can also act as avirulence factors if the plant has evolved resistance proteins which may serve to “guard” or monitor changes which occur on the virulence targets of these effector proteins [45]. In total 55% of these small predicted secreted proteins (<200 aa's) so far have EST support. In addition, homologues of the bona fide effectors ECP2 and ECP6 from the exclusively tomato leaf apoplast infecting species *Cladosporium fulvum* have been identified. Very interestingly 10 of the predicted *M. graminicola* secreted protein possess a Y/F/WxC motifs [32] located in close proximity to the predicted signal peptide sequence. Of these 5 were also very cysteine rich. The function(s) of these motifs, originally identified in abundance in the genome of the haustorium forming and barley leaf infecting ascomycete species *Blumeria graminis* f. sp *hordei*, is not yet known.

This study established a baseline for further analyses which ideally should focus on the changes in gene expression throughout infection, on direct proteomics approaches to verify their predicted secretion and on refined evolutionary analyses. The large number of non-annotated sequences that still remain despite this study poses a further challenge, and therefore exploring their temporal gene expression patterns may provide the first clues to function. Additionally the sequencing of other Dothideomycetes species with different pathogenic and saprophytic lifestyles, other isolates of *M. graminicola* and the subsequent comparative analyses should reveal the repertoire of species -specific secreted proteins found in most *M. graminicola* isolates and those also found in very closely related species. An example of the latter would be the grass infecting species currently termed S1 which also has some ability to infect wheat leaves [46]. These types of studies on highly related species and/or other *M. graminicola* isolates will also reveal the more flexible parts of the *M. graminicola* secretome and which parts of the repertoire of predicted effector proteins exhibit the greatest sequence differences between isolates and/or species.

## Materials and Methods

All protein sequence information can be retrieved from JGI (http://genome.jgi-psf.org/pages/search-for-genes.jsf?organism=Mycgr3) using the unique numerical identifier.

### Bioinformatic analyses of the secretome

#### Stage 1

Version 2 of the *M. graminicola* genome was downloaded from the JGI genome portal (http://genome.jgi-psf.org/Mycgr3/Mycgr3.download.html). The prediction of the refined *M. graminicola* secretome was based on the procedure described by Muller and colleagues [47] for *U. maydis*. We developed an automated secretome prediction pipeline based on this procedure using bash shell, awk and python scripts on a PC running Red Hat Enterprise Linux 5.2. Initially all proteins with a Target P Loc = S (TargetP v1.1; http://www.cbs.dtu.dk/cgi-bin/nph-sw_request?targetp) or a Signal P D-score = Y (SignalP v3.0; http://www.cbs.dtu.dk/cgi-bin/nph-sw_request?signalp) were combined [48], [49]. These were then scanned for transmembrane spanning regions using TMHMM (TMHMM v2.0; http://www.cbs.dtu.dk/cgi-bin/nph-sw_request?tmhmm) and all proteins with 0 TMs or 1 TM, if located in the predicted N-terminal signal peptide, were kept. GPI-anchor proteins were predicted by big-PI (http://mendel.imp.ac.at/gpi/cgi-bin/gpi_pred_fungi.cgi) [50]. ProtComp was also used to predict localization of the remaining proteins using the LocDB and PotLocDB databases (ProtComp v8.0; http://www.softberry.com).

#### Stage 2

WoLF PSORT analysis was done using “runWolfPsortSummary fungi” in the WoLF PSORT v0.2 package, which estimates localisation sites with a sensitivity and specificity of approximately 70% [51]. All proteins predicted with an extracellular score >17 were kept in the final secretome dataset. The selection of this ‘cut-off’ point was tested using a range of experimentally verified secreted fungal proteins from other phytopathogens (Table S2 and Tab 23 in File S1). An extracellular score >17 had previously been used to refine the prediction of the secretome for *Fusarium graminearum* [21] and the results obtained agreed well (68%) with the available proteomics datasets obtained for the *in planta* and *in vitro* secretome of this fungus [52]. PFAM analysis was done using the PFAM database (ftp://ftp.ncbi.nih.gov/pub/mmdb/cdd/) and the rpsblast program in the NCBI blast+ software package (ftp://ftp.ncbi.nlm.nih.gov/blast/executables/blast/). The number of cysteine residues within the mature peptide and the search for degenerative YxC motifs were computed using custom python scripts. The number of internal sequence repeats was found using RADAR (http://www.ebi.ac.uk/Tools/Radar/) [53]. The detection of RNA transcripts for the genes of interest was explored by BLASTN analysis (e-100) of the 13 designated EST libraries available from the JGI website (http://genome.jgi-psf.org/Mycgr3/Mycgr3.download.ftp.html).

### Analysis of chromosome location alongside other key features of the *M. graminicola* genome

To inspect the position of individual genes on the 21 *M. graminicola* chromosomes (Figure 2), the MgraMap tool was downloaded from www.OmniMapFree.org which displays a map of the complete *M. graminicola* genome (JGI version 2.0) [12]. The MgraMap was used according to methods described elsewhere [54].

### Comparative analysis of the refined *M. graminicola* secretome

For the detailed follow up analyses, only proteins in the refined secretome from Stage 2 were used. The *M. graminicola* secretome was compared with 90 proteomes from other fungal, oomycete and plant pathogenic nematode species genomes, varying in host range, tissue specificity and lifestyle as well as several exclusively saprophytic species (Table S1). The fungal and oomycete genomes and their predicted gene repertoires were downloaded from either the BROAD or JGI websites or from species specific websites maintained by various research communities. For the comparative analyses, the conservation, absence or expansion of the genes coding for the *M. graminicola* secreted proteins was explored by BLASTP analysis, determined at two levels of confidence, p<e^−5^ and p<e^−40^.

### Genes coding for proteins with a known function

The total putative secreted proteins related to plant polysaccharides degradation found in *M. graminicola* was predicted using annotation based on the protein family classification from the CAZy – Carbohydrate Active Enzyme website (http://www.cazy.org/). All proteins from the four classes of proteins from the CAZy catalogue (Glycoside Hydrolases, Glycosyl Transferases, Polysaccharide Lyases and Carbohydrate Esterases) were identified initially throughout the genome by a keyword search on the genome browser (http://genome.jgi-psf.org/pages/search-for-genes.jsf?organism=Mycgr3) and then selected for secreted proteins based on a comparison against the predicted secretome. Finally, a manual annotation based on BLASTP analysis against the non-redundant protein database and CAZy classification was done for each protein.

### Data obtained from the JGI and displayed in several of the Supplementary files

For completeness and to assist in inter- sequence comparisons, in each row of Tabs 1 through 6 in File S1, the following information is given in the following columns: (A) protein ID, (B) gene name ID, (C) effector homologue, (D) chromosome location, (E) chromosome position, (F) coding strand, (G) predicted in frozen gene model, (H) predicted in filtered gene model and (I) number of introns predicted.

## Supporting Information

Figure S1 **The distribution of genes encoding secreted peroxidases/chloroperidases (PFAM01328) in the** ***M. graminicola*** **genome.**
(PPTX)

Table S1 **The list of species used for the various comparative analyses.**
(XLSX)

Table S2 **Experimentally verified** s**ecreted proteins related to pathogenicity and virulence, present in other plant pathogenic fungi.**
(DOC)

File S1 **List of all proteins used for analysis in this study.**
(XLSX)
