# Supplementary material for: Defining the Predicted Protein Secretome of the Fungal Wheat Leaf Pathogen Mycosphaerella graminicola
Source: PLoS One. 2012 Dec 7;7(12):e49904. doi: 10.1371/journal.pone.0049904 (PMC3517617; doi:10.1371/journal.pone.0049904)
Supplement: Table S2 — Experimentally verified secreted proteins related to pathogenicity and virulence, present in other plant pathogenic fungi. (DOC) [file pone.0049904.s003.doc]

**Table S2 –** Experimentally verified secreted proteins related to pathogenicity and virulence, present in other plant pathogenic fungi.

| **Protein name (ID)** | **Species** | **WoLFPSORT** | **aa1** | **TMs** | **TMpos** | **SignalPpos** | **Method** | **Taxonomyc class** | **References*** |
| --- | --- | --- | --- | --- | --- | --- | --- | --- | --- |
| Avr2 (CAD16675.1) | *Cladosporium fulvum* | Extr=23 | M | 0 | -- | 20-21 | Recovery from apoplastic fluids / Co-immunoprecipitation | Dothideomycete | Rooney et al (2005) |
| Avr4 (CAA69643.1) | *Cladosporium fulvum* | Extr=14 | M | 0 | -- | 18-19 | Recovery from apoplastic fluids/ Affinity precipitation, fluorescence | Dothideomycete | van den Burg et al (2006) |
| Avr4E (AAT28196.1) | *Cladosporium fulvum* | Extr=8 | M | 0 | -- | 22-23 | Purification of filtrates from apoplast fluids | Dothideomycete | Westerink et al (2004) |
| Avr9 (CAA42824.1) | *Cladosporium fulvum* | Extr=22 | M | 1 | 10_32 | 24-25 | Recovery from apoplastic fluids / High-affinity binding assays | Dothideomycete | Kooman-Gersmann et al (1996) |
| Ecp1 (CAA78400.1) | *Cladosporium fulvum* | Extr=26 | M | 0 | -- | 19-20 | Purification of filtrates from apoplast fluids | Dothideomycete | Laugé et al (2000) |
| Ecp2 (CAA78401.1) | *Cladosporium fulvum* | Extr=27 | M | 0 | -- | 18-19 | Purification of filtrates from apoplast fluids | Dothideomycete | Laugé et al (2000) |
| Ecp4 (CAC01609.1) | *Cladosporium fulvum* | Extr=26 | M | 0 | -- | 19-20 | Purification of filtrates from apoplast fluids | Dothideomycete | Laugé et al (2000) |
| Ecp5 (CAC01610.1) | *Cladosporium fulvum* | Extr=24 | M | 0 | -- | 19-20 | Purification of filtrates from apoplast fluids | Dothideomycete | Laugé et al (2000) |
| Ecp6 (ACF19427.1) | *Cladosporium fulvum* | Extr=27 | M | 0 | -- | 18-19 | Recovery from apoplastic fluids / 2D-PAGE | Dothideomycete | Bolton et al (2008) |
| LAC2 (BAM42534.1) | *Colletotrichum orbiculare* | Extr=23 | M | 0 | -- | 19-20 | Accumulation of GFP fluorescence close to the cell surface of the conidia | Sordariomycete | Lin et al (2012) |
| NIS1 (BAL70334.1) | *Colletotrichum orbiculare* | Extr=23 | M | 0 | -- | 19-20 | HA-tagged protein and immunodetection in culture filtrate | Sordariomycete | Yoshino et al (2012) |
| FGL1 (AAQ23181.1) | *Fusarium graminearum* | Extr=27 | M | 0 | -- | 15-16 | Enzymatic activity in culture supernatant | Sordariomycete | Salomon et al (2012) |
| Avr1 (SIX4) (CAJ84000.1) | *Fusarium oxysporum f. sp. lycopersici* | Extr=27 | M | 0 | -- | 17-18 | Extracted from xylem sap / 2-D PAGE | Sordariomycete | Houterman et al (2007) |
| Avr2 (SIX3) (CAJ83999.1) | *Fusarium oxysporum f. sp. lycopersici* | Extr=19 | M | 0 | -- | 19-20 | Extracted from xylem sap / 2-D PAGE | Sordariomycete | Houterman et al (2007) |
| Avr3 (SIX1) (CAE55870.1) | *Fusarium oxysporum f. sp. lycopersici* | Extr=16 | M | 0 | -- | 21-22 | Extracted from xylem sap / 2-D PAGE | Sordariomycete | Houterman et al (2007) |
| SIX2 (CAE55868.3) | *Fusarium oxysporum f. sp. lycopersici* | Extr=25 | M | 0 | -- | 20-21 | Extracted from xylem sap / 2-D PAGE | Sordariomycete | Houterman et al (2007) |
| Avr-Pita1 (AAK00131.1) | *Magnaporthe oryzae* | Extr=6 | M | 0 | -- | 16-17 | Fluorescence fused protein (does not translocate into the infected host cells, but localizes in BIC) | Sordariomycete | Khang et al (2010) |
| BAS1 (ACQ73206.1) | *Magnaporthe oryzae* | Extr=11 | M | 0 | -- | 22-23 | Fluorescent protein translocated into the cytoplasm of invaded host cells | Sordariomycete | Khang et al (2010) |
| BAS2 (ACQ73207.1) | *Magnaporthe oryzae* | Extr=25 | M | 0 | -- | 19-20 | Fluorescence (does not translocate into the infected host cells, but localizes in BIC) | Sordariomycete | Mosquera et al (2009) |
| BAS3 (ACQ73208.1) | *Magnaporthe oryzae* | Extr=19 | M | 0 | -- | 20-21 | Fluorescent fused protein accumulates at host wall crossing points | Sordariomycete | Mosquera et al (2009) |
| BAS4 (ACQ73209.1) | *Magnaporthe oryzae* | Extr=20 | M | 0 | -- | 21-22 | Fluorescent fused protein found in the host apoplast | Sordariomycete | Khang et al (2010) |
| Slp1 (XP_003717420.1) | *Magnaporthe oryzae* | Extr=26 | M | 0 | -- | 16-17 | Fluorescent fusion proteins in the apoplastic space | Sordariomycete | Mentlak et al (2012) |
| MC69 (EHA46146.1) | *Magnaporthe oryzae* | Extr=18 | M | 1 | 5_24 | 16-17 | Fluorescence (does not translocate into the infected host cells, but localizes in BIC) | Sordariomycete | Saitoh et al (2012) |
| PWL1 (AAA80239.2) | *Magnaporthe oryzae* | Extr=20 | M | 0 | -- | 23-24 | Fluorescence (does not translocate into the infected host cells, but localizes in BIC) | Sordariomycete | Khang et al (2010) |
| PWL2 (AAA91019.1) | *Magnaporthe oryzae* | Extr=18 | M | 1 | 7_26 | 21-22 | mCherry::NLS fusion protein exhibits fluorescence in nuclei of host cell | Sordariomycete | Khang et al (2010) |
| Ptr ToxA (AAB70095.1) | *Pyrenophora tritici-repentis* | Extr=25 | M | 0 | -- | 16-17 | Immunolocalization and green fluorescent protein tagged protein found in the chloroplasts | Dothideomycete | Manning and Ciuffetti (2005) |
| Ptr ToxB (AAN39056.1) | *Pyrenophora tritici-repentis* | Extr=24 | M | 1 | 10_32 | 23-24 | Immunodetection (Western blotting) protein from culture filtrates | Dothideomycete | Cao et al (2009) |
| NIP1 (AAA86496.1) | *Rhynchosporium secalis* | Extr=23 | M | 0 | -- | 20-21 | High-affinity binding assays | Ascomycete | van’t Slot et al (2007) |
| NIP2 (AFI43935.1) | *Rhynchosporium secalis* | Extr=23 | M | 0 | -- | 16-17 | Purification from culture filtrates | Ascomycete | Wevelsiep et al (1993) |
| NIP3 (AFI43936.1) | *Rhynchosporium secalis* | Extr=6 | M | 0 | -- | 17-18 | Purification from culture filtrates, measurement of activity on plant plasma membrane | Ascomycete | Wevelsiep et al (1993) |
| SnToxA (ABD85141.1) | *Stagonospora nodorum* | Extr=24 | M | 0 | -- | 16-17 | Culture filtrate and purification | Dothideomycete | Liu et al (2006) |
| SnTox1 (AEX93345.1) | *Stagonospora nodorum* | Extr=16 | M | 0 | -- | 17-18 | Culture filtrate and purification | Dothideomycete | Liu et al (2012) |
| SnTox3 (ACR78113.1) | *Stagonospora nodorum* | Extr=23 | M | 0 | -- | 20-21 | Culture filtrate and purification | Dothideomycete | Friesen et al (2008) |
| Pit2 (XP_757522 .1) | *Ustilago maydis* | Extr=18 | M | 0 | -- | 25-26 | m-Cherry fusion protein Fluorescence found in apoplast post plasmolysis | Ustilaginomycete | Doehlemann et al (2011) |
| Pep1 (XP_758134.1) | *Ustilago maydis* | Extr=27 | M | 0 | -- | 25-26 | *In situ* immunodetection and fluorescence of tagged proteins | Ustilaginomycete | Doehlemann et al (2009) |

(*) Find the complete references in the Supplementary file 23.
